# Body Mobility and Attention Networks in 6- to 7-Year-Old Children

**DOI:** 10.3389/fpsyg.2021.743504

**Published:** 2021-10-27

**Authors:** Joëlle Rosenbaum, Jean-Michel Hascoët, Isabelle Hamon, Arthur Petel, Sébastien Caudron, Hadrien Ceyte

**Affiliations:** ^1^Université de Lorraine, DevAH, Nancy, France; ^2^Université de Lorraine, CHRU, Maternité Régionale, Département de Néonatologie, Nancy, France; ^3^Université Grenoble Alpes, Université Savoie Mont Blanc, CNRS, LPNC, Grenoble, France

**Keywords:** body mobility, posture, attention networks, school-aged children, alertness, orienting, executive control

## Abstract

Learning in 6- to 7-year-old children is strongly influenced by three functions of attention: alertness, orienting, and executive control. These functions share a close relationship with body mobility, such as the posture adopted or a request to stay still during tasks. The aim of this study (ClinicalTrials.gov) was to analyze the influence of body posture (standing versus sitting) and the influence of these imposed postures compared to a free body mobility on attention functions in 6- to 7-year-old children. Twenty-one children (11 girls) with a mean age of 6.7±0.6years performed the Attention Network Test for Children in three-body mobility conditions: sitting still, standing still, and free to move. Three attentional scores were calculated which would separately reflect performance of alertness, orienting, and executive control. Overall, no difference in alertness performance was found between the three bodily mobility conditions. In addition, our results suggest a general poor orienting performance in children, whatever the body mobility condition, which might be related to their young age. Finally, children improved their executive control performance when they stood still, probably due to an improvement in arousal and mental state.

## Introduction

The ages of 6 and 7 years old are crucial for fundamental learnings of children. Indeed, the end of kindergarten and the beginning of primary school is marked by the discoveries of reading, writing, and mathematics. Concomitantly, this period corresponds to an important step in cognitive development characterized by quantitative and qualitative changes in several cognitive processes. Indeed, besides an important improvement in processing speed (Kail and Ferrer, 2007), efficient cognitive strategies may emerge in children aged 6 to 7, such as “sorting” or “active refreshing” strategies improving short-term and working memory performance (Schneider et al., 2004; Camos and Barrouillet, 2011). These children start also to use a more proactive cognitive control (Lucenet and Blaye, 2014; Gonthier et al., 2019), by preparing in advance for foreseeable demands of tasks, which is known to require more mental efforts but also to be more efficient for minimizing the effects of interfering information (Chevalier, 2015). These changes are accompanied by an important increase in attention performance (Mezzacappa, 2004). The development literature has consistently shown that executive functions and attention components play an important part in children’s academic achievement (Cartwright, 2012; Franceschini et al., 2012; Kim et al., 2013; Hassinger-Das et al., 2014; Kent et al., 2014; Meixner et al., 2019; Valdois et al., 2019).

Posner and Petersen (1990) identified three functions of attention named alertness, orienting, and executive control, all sustained by distinct neural networks. Thus, alertness refers to the elaboration and maintenance of an optimal level of vigilance to be ready to achieve a task or respond to a stimulus (Raz and Buhle, 2006; Petersen and Posner, 2012; Rueda and Posner, 2013; Barra et al., 2015). It can be divided into tonic alertness [i.e., a general cognitive control of arousal during the task (Raz and Buhle, 2006)] and phasic alertness [i.e., a more transient level of readiness to respond (Petersen and Posner, 2012)]. Alertness is controlled by a norepinephrine pathway from the locus coeruleus to frontal and parietal regions. Orienting is related to the ability to enhance the processing of specific information from sensory input by shifting attention to a particular object, event, or location (Waszak et al., 2010; Barra et al., 2015; Mullane et al., 2016). It is controlled by a ventral network, including the temporo-parietal junction and the ventral frontal cortex, and a dorsal network including the frontal eye field and the intraparietal sulcus (Petersen and Posner, 2012). The third function identified by Posner and Petersen (1990), the executive control, refers to error detection, monitoring, and resolution of conflict in goal setting [e.g., during thoughts, feelings, and responses (Petersen and Posner, 2012; Rueda and Posner, 2013)]. In particular, executive control allows the inhibition of irrelevant response tendencies or distracting stimuli to focus on the task demand (Raz and Buhle, 2006). Two dopaminergic networks, the fronto-parietal and cingulo-opercular networks, are mainly involved in these executive processes. The efficiency of the three functions of attention may be evaluated by a single task with the Attention Network Test (Fan et al., 2002). Notably, Rueda et al. (2004) developed a version of this test for children (ANT-C) to study the development of these functions during childhood.

From a developmental point of view, phasic alertness following a visual warning signal gradually improves until 6years of age, with a second developmental phase during early adolescence (Mezzacappa, 2004; Rueda et al., 2004; Federico et al., 2017; Suades-González et al., 2017; Lewis et al., 2018). When an auditory warning signal was used, an improvement of phasic alertness until 12years of age was evidenced, especially between 7 and 9years (Pozuelos et al., 2014; Mullane et al., 2016). Tonic alertness significantly increases in preschoolers (Rueda and Posner, 2013) but may continue to develop until adolescence for monotonous task, with a significant improvement between 6 and 8–9years old (Lin et al., 1999; Kanaka et al., 2008; Lewis et al., 2017). In contrast, the automatic, exogenous orienting toward targets may be almost mature around the age of 6years (Rueda et al., 2004), even if an improvement between 5 and 7years old was shown (Mezzacappa, 2004). Endogenous orienting guided by internal intentions (e.g., following an arrow pointing toward the target location) and the attentional disengagement and reorienting (following invalid spatial information) continue to develop after 6years old, at least until late childhood (Konrad et al., 2005; Wainwright and Bryson, 2005; Iarocci et al., 2009; Waszak et al., 2010; Pozuelos et al., 2014; Johnson et al., 2020). Finally, it is well recognized that executive functions and abilities of children to resolve conflict strongly improved during early childhood (Carlson, 2005; Diamond, 2006; Garon et al., 2008). Nevertheless, depending on the tasks used, an improvement of executive control during moderate and late childhood was highlighted (Best and Miller, 2010). Notably, studies using ANT-C and Flanker tasks showed a development of executive control between 4 and 9years old (Mezzacappa, 2004; Rueda et al., 2004; Checa et al., 2014; Pozuelos et al., 2014; Mullane et al., 2016; Lewis et al., 2018). Its development may extend throughout adolescence because 15-year-old teenagers are not yet at the adult level (Waszak et al., 2010). Therefore, 6 to 7years of age is a crucial period for attentional development during which alertness is not fully mature, the development of exogenous orienting may be completed, and executive control strongly improves. Many environmental factors may influence attentional development and performance at these ages, such as socioeconomic status (Mezzacappa, 2004) or training intervention (Rueda et al., 2005).

Interactions between postural and cognitive, especially attention, activities were studied in adults (Dault et al., 2001; Mitra and Fraizer, 2004) and in children (Olivier et al., 2010; Fabri et al., 2017). These studies were done with the dual-task paradigm: cognitive and postural respective performances are compared between the separate and the concurrent execution of one postural and one cognitive task. Another method consists in observing how the increase in the difficulty level of one task affects the performance in both tasks (Fraizer and Mitra, 2008). The most common explanation for the results observed is that postural and cognitive activities share a set of limited central processing resources which results in decreased performance of one or both tasks if the amount of available resources is exceeded (Woollacott and Shumway-Cook, 2002). However, this conception is not sufficient for explaining the great variety of results observed (Bonnet and Baudry, 2016). Indeed, some cognitive performances may improve in more demanding postures (Barra et al., 2015) and some cognitive activities cause a decrease in body sway – interpreted as a better postural performance – by diverting attention focus from postural control and increasing automaticity (Richer et al., 2017).

Thus, an alternative and more ecological conception was proposed (Stoffregen et al., 1999, 2000, 2007). Postural activity would be functionally integrated with suprapostural activities defined as the “cognitive activities superordinate to the control of posture” (Stoffregen et al., 1999), that is, cognitive activities other than postural control. For example, postural stabilization may occur during precise perceptive tasks in order to improve visual performance (Stoffregen et al., 2007). Other authors also speculated on a functional role of body sway, interpreted as a self-generated body mobility in order to increase the organism’s arousal (Stins and Beek, 2012; Ceyte et al., 2014). Therefore, postural and suprapostural tasks influence each other and the performance during dual tasks strongly depends on the posture and the cognitive function studied (Stoffregen et al., 2007; Abou Khalil et al., 2020). The differences in body sway between a reading and a counting backward task in children aged 8–10 suggest that postural activity may be modulated to facilitate suprapostural task from childhood (Blanchard et al., 2005).

From the first grade of elementary school, the preferential posture at school (in many cultures) remains sitting still, without moving, because body mobility is supposed to disturb their classwork. However, the use of school desks allowing to freely sit down or stand up did not decrease attention performance (Koepp et al., 2012; Aminian et al., 2015; Wick et al., 2018). On the contrary, teachers and children reported that using these desks improved their attention performance (Blake et al., 2012; Aminian et al., 2015; Verloigne et al., 2018; Wick et al., 2018). Therefore, changing posture during lessons may improve children attention. In adults, an increase in alertness (Caldwell et al., 2003; Barra et al., 2015) and to a lesser extent in executive control performance (Rosenbaum et al., 2017; Smith et al., 2019, but see Caron et al., 2020; Kang et al., 2021) were observed by adopting a standing compared to a sitting posture. These improvements may derive from neurophysiological modulations occurring when standing (Tulen et al., 1999; Hennig et al., 2000; Caldwell et al., 2003; Thibault and Raz, 2016) which may increase the level of general and cortical arousal (Barra et al., 2015; Smith et al., 2019). In addition, the lack of difference between sitting and standing for orienting suggests different effects of body posture depending on the attentional function studied (Barra et al., 2015).

This influence of postural and motor systems on higher-cognitive processes supports that mind is “embodied” in bodily experiences. In the theoretical conception of embodied cognition, actions and sensorimotor experiences determine our major cognitive processes (Brouillet, 2019). The crucial role of bodily experiences is also increasingly highlighted for child development and cognition (Marmeleira and Duarte Santos, 2019). For example, bodily and “embodied” activities may enhance mathematical or scientific learnings (Dackermann et al., 2017; Mavilidi et al., 2017). Interestingly, the improvement of working memory performance when children walk (Schaefer, 2019), especially at an un-imposed speed (Schaefer et al., 2010), and their increased motor activity level during cognitive demanding tasks (Patros et al., 2017) may suggest a functional role of spontaneous body mobility on high-cognitive processes. The improvement of children’s tonic alertness (de Greeff et al., 2018) and executive control (Janssen et al., 2014; de Greeff et al., 2018) after acute physical activities suggests that body mobility may also influence attentional functions.

The aim of this study was to analyze the influence of body posture (standing versus sitting) and the influence of these imposed postures compared to a free body mobility on attentional functions in 6- to 7-year-old children. We hypothesized that alertness and executive control, but not orienting, performance improves with standing compared to sitting. Moreover, with a free to move condition, compared to the instruction to stay still, we expected that children would increase their body mobility, which would improve the efficiency of their attentional functions.

## Materials and Methods

### Participants

Twenty-five children born at term (39.3±1.3weeks of gestation) with a mean age of 6.7±0.6years (12 girls and 13 boys) were recruited for this study. Among them, three children did not fully complete the experiment (one due to technical issues and two due to a fatigue effect), and one child failed to respect the instructions for the task (51.7% errors). Finally, 21 children (mean age=6.7±0.6years, 11 girls, 10 boys) were included in the analysis. Before the experimental session, the children underwent a preliminary clinical examination conducted by a certified trained pediatrician which reported their body weight, height and verified that they had normal hearing and normal or corrected-to-normal vision. The pediatrician also verified by parental interview and clinical examination that the child had no neurological, cognitive, developmental, or motor disorder preventing the execution of the tests. The general IQ of children was not assessed but it was verified that each child was in his expected grade and no child needed special education support. Four children were preschoolers, 10 children were in grade 1, and 7 children were in grade 2. The parents of these children were mostly in couple (20 parents in couple, 1 no reported), and both parents worked [including executives and intellectual professionals (8 mothers and 12 fathers), intermediaries (12 mothers and 7 fathers), artisans/shopkeepers (1 mother and 1 father), and employees/workers (1 father; Institut National de la Statistique et des Etudes Economiques, 2003)].

Parents and children were informed about the experiment using an information leaflet displayed in schools and Lorraine University. All parents gave their written consent and all children their oral consent.

### Materials and Experimental Design

The materials and method of this experiment were described previously (Ceyte et al., 2018) and were recapitulated below.

Children performed the ANT-C generated by E-Prime software (version 3.0 professional; Psychological Software Tools®, Sharpsburg PA, United States) using a head-mounted display (iWear Video Headphones, The Way In®, Vuzix Corporation, NY, United States) equipped with two 24-bit color visual displays. The visual displays covered a diagonal field of view of 55 degrees with a resolution of 1,280×720 pixels to keep the distance between the children’s eyes and targets constant across the different experimental conditions.

#### ANT-C Task

The ANT-C task was previously used to evaluate attention performance of children aged 6 to 7years (Rueda et al., 2004, see also Ishigami and Klein, 2015). Using their preferred hand, children had to click on the right or left button of a mouse as fast as possible to indicate the facing direction (right or left) of a yellow target fish. This target fish subtended 1.6deg. of the visual angle and could appear 1deg. above or 1deg. below a black central fixation cross. Each trial followed the same pattern (Supplementary Figure S1).

The trial started by a fixation period of a random duration from 400 to 1,600ms.

Then, the target fish could appear following different precueing conditions. In 25% of trials, the fish suddenly appeared without warning signal (“no cue” trials) 600ms after the fixation period. In 75% of trials, one of three equiprobable warning cues could appear 400ms before the occurrence of the target: (i) a “double cue” formed by two asterisks appearing 1deg. above and below the fixation cross, which alerted to the imminent appearance of the fish, (ii) a “center cue” (one asterisk) appearing at the place of the central fixation cross, and (iii) a “spatial cue” (one asterisk) appearing at the location of the upcoming target fish which oriented attention toward the target’s location. The cue appeared during 150ms.

The target fish then appeared within a limit of 2,500ms. The target could appear alone or flanked by either 4 congruent fishes facing the same direction or 4 incongruent fishes facing the opposite direction (all fishes subtended a total of 8.8deg. of the visual angle). Children had to indicate the facing direction of the central fish.

Finally, after another fixation period of 3,500ms minus the child response time, the next trial began.

#### Body Mobility Conditions

Children performed the ANT-C in three-body mobility conditions. They received the oral instruction to *stay still* either in a sitting posture on a chair with a seatback and without armrest (*sitting still* condition) or in a standing posture with feet apart at children preferred spacing (*standing still* condition). Thus, before the children performed the ANT-C in the sitting still or in the standing still posture, the following was explained: “*You have to stay as still as possible, like a statue, without moving your head, your arm, or your leg. In addition, you have to catch the fish as fast as possible*.” They were asked to adopt the still posture for a few seconds to confirm that they understood the instruction. Finally, in a *free to move* condition, children could adopt the posture(s) of their choice and move as they wanted. The following was explained to the children: “*You can take the position you want and change position or move as you want. For example, you can lie down, sit down, or stand up. More important is to catch the fish as fast as possible. Set yourself up how you want to start the game*.” Each experimental session was video-recorded to analyze the children’s mobility thoroughly *a posteriori*.

### Procedure

Each child performed a practice block of 12 trials of the ANT-C in a non-restricted sitting posture to ensure they understood the instructions of the ANT-C. They then completed an experimental block of the ANT-C in each body mobility condition. One experimental block included 48 trials [4 cue types (no, double, center, spatial)×3 target types (alone, congruent, incongruent)×2 fish locations (above, below the fixation cross)×2 fish orientations (to the left, to the right)] and lasted approximately 4min 30s. The three experimental blocks (one experimental block per mobility condition) were randomized in blocks of 6 so that the same number of children eventually adopted the sitting still, the standing still and the free to move condition in first position. Thirty seconds of rest were given in the middle of each block and 3min of rest between each body mobility condition. The experiment lasted approximately 45min.

### Data Analysis

#### Movements of Children During the ANT-C

Observation from video analysis was made by J.R. and H.C. who independently coded the moving body parts and duration of movements using a standardized observation grid. A second observation was made by J.R. 1month after the end of the first observation to validate the reliability of the first observation. Four types of movement were defined: movement of the lower limbs (including leg movement, feet movement, and walking), upper limbs (arm movement, hand movement, and scratching), and head, as well as changes in position for the free to move condition only. The mean duration of the three observations was computed for each type of movement. To estimate the amount of movement depending on the body segment that moved, each movement duration was weighted by an estimation of the segment mass that moved (percent of body mass) using the mathematical equation from Jensen (1989) and the child’s age. For each child, these weighted durations were added and normalized to 1min to provide the global amount of movement during the test.

The agreement and reliability between the three observations were performed on ratings of movement of the upper limbs (by grouping arm and hand movements) and lower limbs (by grouping foot and leg movements) because the children mostly moved these body parts. For the detection of movement (coded 0 when 0 movement or one single 1-s movement was reported and 1 otherwise), the mean agreement between the 3 observations was *Cohen’s kappa k*=0.56 for the upper limbs (range: 0.50 to 0.63) and k=0.59 for the lower limbs (range: 0.44 to 0.66). The absolute percentage of agreement was 81% in mean for the upper limbs (range: 78 to 86%) and 82% for the lower limbs (range: 75 to 87%). Spearman *ρ* was performed on movement duration of the lower and upper limbs, excluding ratings where 0 movement was reported in all observations (in order to limit the number of ties). Max-min method was used to take into account the remaining ties (Amerise and Tarsitano, 2015). The reliability between the three observations was *ρ*=0.65 in mean for the lower limbs (range: 0.57 to 0.77, all *p*<0.05) and *ρ*=0.80 in mean for the upper limbs (range: 0.77 to 0.86, all *p*<0.05).

To confirm that children respected the instruction to stay still or to move more in the free to move condition, Friedman tests and Wilcoxon matched-pair signed ranks tests with appropriate Bonferroni correction were performed on the global amount of movement because the data were not normally distributed (Shapiro-Wilk tests, *p*<0.05). Effect size was determined by Kendall’s W coefficient (*W*) for the Friedman test and rank biserial correlation (r) for the Wilcoxon matched-pair signed ranks test (Tomczak and Tomczak, 2014). Medians (*Med*) and interquartile ranges (*IQRs*) are presented.

#### Children’s Attention Networks

Children success or failure and their RT were recorded for each trial. This allowed to calculate, for each child, the success rate and median reaction time for correct responses (M_RT-c_) for different cue and target conditions (Rueda et al., 2004).

One score for each attention function was then calculated using M_RT-c_. An *alerting score* was calculated by subtracting M_RT-c_ for double cue trials from M_RT-c_ for no cue trials, that is, for trials in which the children were, respectively, alerted and not alerted to the imminent appearance of the fish. An *orienting score* was calculated by subtracting M_RT-c_ for spatial cue trials from M_RT-c_ for center cue trials, that is, for trials in which the cue oriented the attention, respectively, toward the fish location and a central location. Finally, a *conflict score* was calculated by subtracting M_RT-c_ for congruent trials from M_RT-c_ for incongruent trials, that is, for trials in which the flanking fishes provided, respectively, the same and conflicting directions compared to the target fish.

Statistical analyses were performed with planned contrasts computed (1) for each attention score (alerting score, orienting score, and conflict score) and (2) for the M_RT-c_ of the different cue and target conditions. Contrasts and coefficients are presented in Table 1. The three first contrasts let to confirm the alerting effect of a warning cue (double cue) compared to no cue on M_RT-c_ (Ac1), the orienting effect of a spatial cue compared to a center cue (Ac2), and the conflict effect of incongruent targets compared to congruent targets (Ac3). The other contrasts enable us to compare attention scores between the sitting still condition and the standing still condition (A1, O1, and C1), and between the imposed postures (both sitting and standing still) and the free to move condition (A2, O2, and C2).

**Table 1:** Sets of coefficients used for contrasts in the experiment.

Contrast		Sitting	Standing	Free		Sitting	Standing	Free
*Ac1*	No cue	1	1	1	Double cue	–1	–1	–1
*Oc1*	Center cue	1	1	1	Spatial cue	–1	–1	–1
*Cc1*	Incongruent	1	1	1	Congruent	–1	–1	–1
*A1*	Alerting score	1	–1	0				
*A2*	Alerting score	1	1	−2				
*O1*	Orienting score	1	–1	0				
*O2*	Orienting score	1	1	−2				
*C1*	Conflict score	1	–1	0				
*C2*	Conflict score	1	1	−2				

Data are presented as mean (*M*) and standard deviation (*SD*). The significance threshold was set at 0.05, and standard error (SE) is presented for each significant value. Effect sizes were analyzed by *Cohen’s d*. Analyses were performed in STATISTICA® software (TIBCO Software, Inc., Tulsa, OK, United States).

## Results

### Movements of Children During the ANT-C

**Figure 1:**
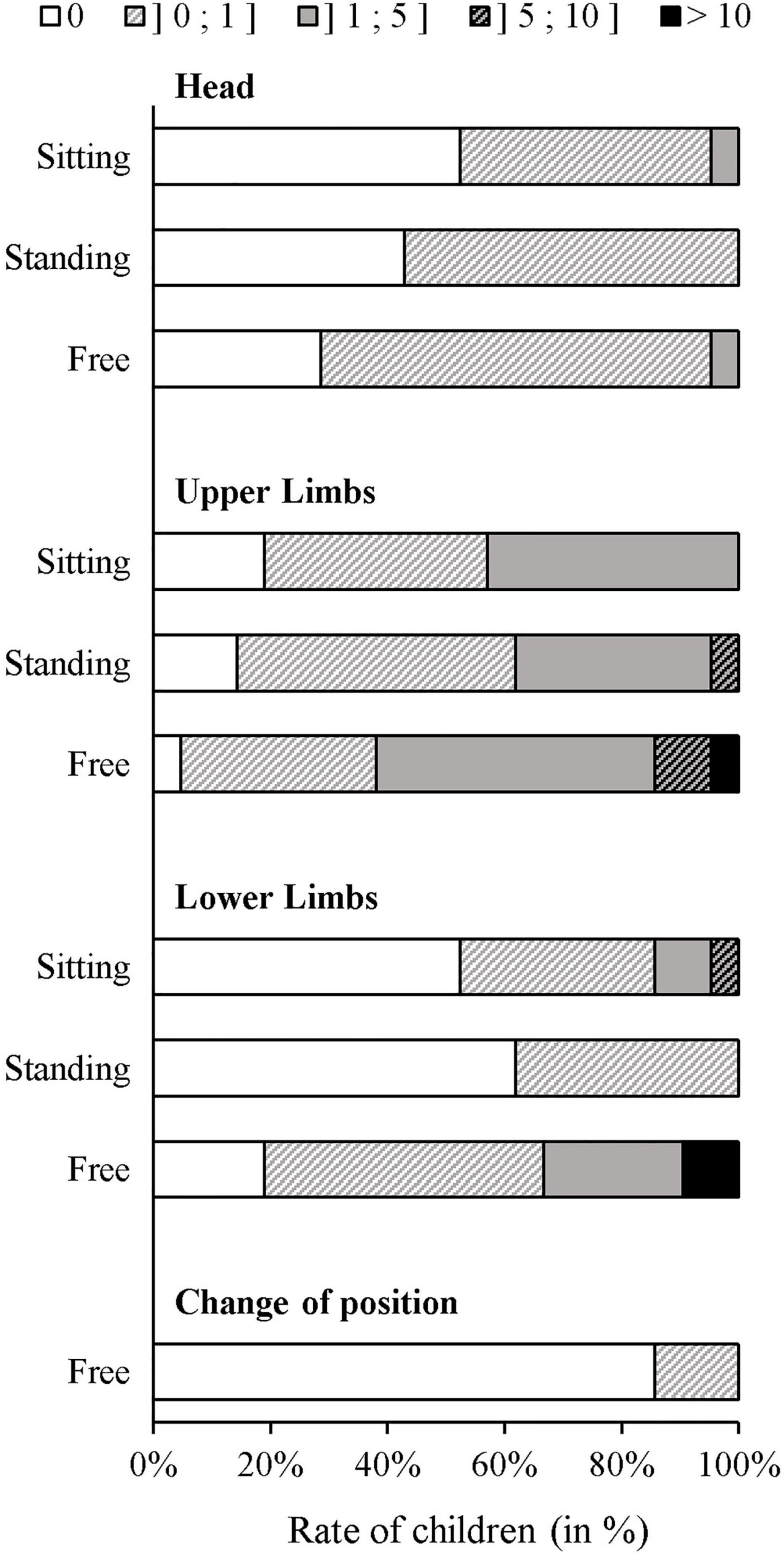
Proportion of children not moving (0), moving between 0 and 1, 1 and 5, 5 and 10 and more than 10s per minute for each type of movement in each body mobility condition.

As indicated in Figure 1, for all types of movement and all body mobility conditions, the majority of children moved between 0 and 5s per minute. Regarding the free to move condition, all children seated down at least one time (among them 3 kneeled and 4 cross-legged) and 11 children exclusively seated down when they performed the ANT-C. Six children adopted at least one time a standing posture and the same number lied down (2 lied on the stomach, the others on the back) at least one time.

We found a significant effect of body mobility condition on the global amount of movement, *χ*^2^(21,2)=14.19, *p*=0.001, *W*=0.34. The Wilcoxon matched-pair test revealed a greater global amount of movement in the free to move condition (*Med*=0.16, *IQR*=0.50) than in the sitting still condition (*Med*=0.07, *IQR*=0.15), *Z*(20)=3.22, *p*=0.001, *r*=0.80 and the standing still condition (*Med*=0.07, *IQR*=0.13), *Z*(20)=3.25, *p*=0.001, *r*=0.81. No difference was found between the sitting still and standing still conditions, *Z*(20)=0.37, *p*=0.70.

### ANT-C Performance

The general success rate, based on the proportion of correct responses, was high and similar between the sitting still (*Med*=95.8%, *IQR*=5.2%), standing still (*Med*=95.8%, *IQR*=4.2%), and free to move conditions (*Med*=93.8%, *IQR*=4.2%). Friedman analysis revealed no effect of the body mobility condition on success rate, *χ*^2^(21,2)=1.1, *p*=0.58. Therefore, further analyses were performed using children’s M_RT-c_.

#### Alerting, Orienting, and Conflict Effects of Cue and Target

M_RT-c_ for the different cue and target conditions in each body mobility condition are summarized in Table 2, and the contrasts used are summarized in Table 1 (Ac1, Oc1, Cc1). Children responded significantly faster in double cue trials (*M*=917, *SD*=248) than in no cue trials (*M*=997, *SD*=194), *t*(20)=3.77, *p*=0.002, *SE*=64, *d*=0.36 (Ac1), confirming the alerting effect induced by a warning cue on M_RT-c_. Conversely, no difference was found between the center cue (*M*=916, *SD*=200) and spatial cue trials (*M*=927, *SD*=200), *t*(20)=0.61, *p*=0.51 (Oc1), suggesting that children did not orient their attention significantly faster with spatial information on the upcoming target. Finally, children responded significantly faster in congruent trials (*M*=910, *SD*=193) than incongruent trials (*M*=1,019, *SD*=235), *t*(20)=7.15, *p*<0.001, *SE*=46, *d*=0.51 (Cc1), confirming the conflict effect induced by incongruent targets on M_RT-c_.

**Table 2:** Mean of median reaction time (in ms) for different cue and target conditions in each body mobility condition.

	Sitting	Standing	Free
Double cue	911 (260)	905 (267)	934 (228)
No cue	993 (205)	1,002 (175)	996 (209)
Spatial cue	916 (206)	921 (199)	888 (218)
Center cue	902 (191)	902 (210)	944 (205)
Congruent	894 (199)	906 (177)	930 (211)
Incongruent	1,045 (260)	1,001 (214)	1,010 (238)

Standard deviation are in parentheses.

#### Effects of Body Mobility Condition on Attention Score

**Figure 2:**
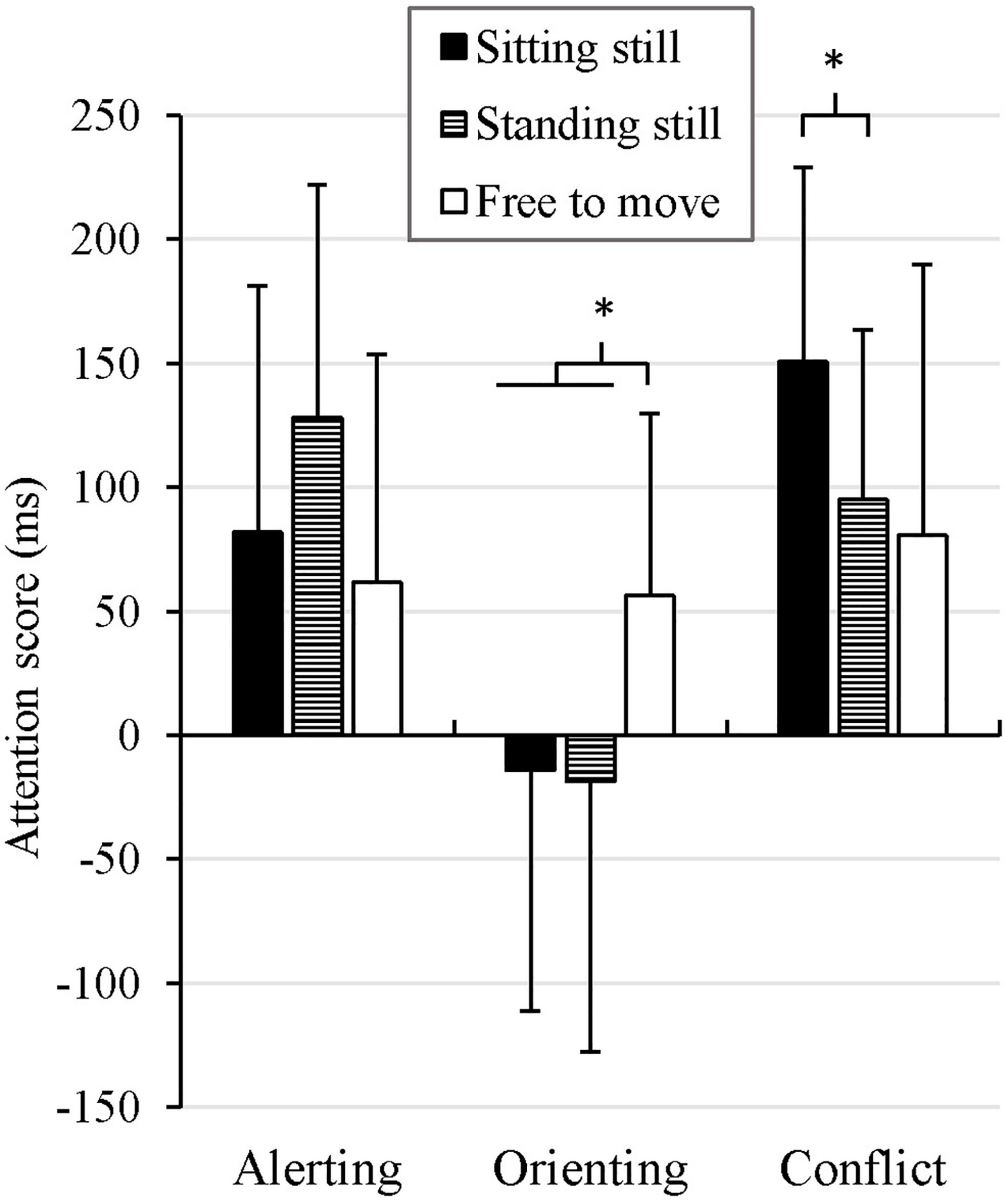
Alerting score, orienting score, and conflict score in each body mobility condition. Error bars represent the mean absolute difference. ^*^p<0.05.

The three attention scores in each body mobility condition is summarized in Figure 2, and the contrasts used are summarized in Table 1 (A1, A2, O1, O2, C1, and C2).

##### Alerting Score

One value (in the standing still condition) was higher than *Mean+3 Standard Deviation* so it was replaced using the K-nearest neighbor algorithm. No difference was found in the alerting score between the sitting still (*M* = 82ms, *SD*=115) and standing still conditions (*M* = 128ms, *SD*=133), *t*(20)=−1.52, *p*=0.14 (A1). Furthermore, no difference was found between the imposed postures (*M*=105ms, *SD*=125) and the free to move condition (*M*=62ms, *SD*=123), *t*(20)=1.46, *p* = 0.16 (A2).

##### Orienting Score

No difference was found in the orienting score between the sitting still (*M*=−14ms, *SD*=113) and standing still conditions (*M*=−18ms, *SD*=139), *t*(20)=0.13, *p*=0.90 (O1). However, the orienting score was significantly larger in the free to move condition (*M*=56ms, *SD*=95) than in the imposed postures (*M*=−16, *SD*=125), *t*(20)=2.24, *p*=0.04, *SE*=65, *d*=0.65 (O2).

##### Conflict Score

The planned contrast revealed a significantly larger conflict score in the sitting still condition (*M*=151ms, *SD*=100) than in the standing still condition (*M*=95ms, *SD*=86), *t*(20)=2.20, *p*=0.04, *SE*=25, *d*=0.60 (C1). No difference was found between the imposed postures (*M*=123ms, *SD*=96) and the free to move condition (*M*=81ms, *SD*=134), *t*(20)=1.34, *p*=0.19 (C2).

## Discussion

The aim of this study was to analyze the influence of body posture (standing versus sitting) and the influence of these imposed postures compared to a free body mobility on alertness, orienting, and executive control in children aged 6 to 7years. Contrary to our hypothesis, children did not significantly improve their alertness, neither when they stood still nor when they moved freely, compared to sitting still. Regarding the function of orienting, as expected, we found no difference between sitting and standing still. A slightly larger orienting score was found when the children were free to move. Nevertheless, in general, children did not respond faster when they were previously informed of where the target would appear. Finally, we expected an improvement in executive control when standing compared to sitting, and without the instruction to stay still. The results showed that children did have improved executive control when standing, but we found no further improvement when they were free to move.

### Insufficient Mobility for Improved Alertness?

We found no difference between sitting and standing still for children’s alertness. These results are different from previous reports in adults (Caldwell et al., 2003; Barra et al., 2015). In the one hand, the influence of adopting a standing posture on the level of alertness may differ between children aged 6 to 7years and adults, as their postural control (Assaiante and Amblard, 1995; Olivier et al., 2008; Micarelli et al., 2020) and their function of alertness (Rueda et al., 2004) are yet not fully developed. In the other hand, perhaps the instruction to stay still prevented any improvement in alertness with the standing posture. Indeed, directing the attentional focus toward postural control (termed internal focus) causes a more conscious regulation of posture, which may interfere with the automatic processes of postural control and may disrupt postural and cognitive activities in adults and children (Olivier et al., 2008; Remaud et al., 2013; Wulf, 2013; Burcal et al., 2014; Jehu et al., 2014; Richer and Lajoie, 2020). In addition, the instruction to stay still may require the inhibition of spontaneous or impulse motor responses in children and motor inhibitory control may develop into late childhood and adolescence depending on the inhibition task (Best and Miller, 2010).

It can be noted that the use of sit-to-stand desks at school, which seem to improve children’s alertness, allows them to change posture as often as they want (Aminian et al., 2015). This may allow them to increase their body mobility compared to a standing still posture. In the present study, children did not improve their alertness when they were free to move. Nevertheless, the majority of children moved relatively little, even without the instruction to stay still. Therefore, perhaps that a greater body mobility may be necessary to improve the children’s alertness. The increase in children’s tonic alertness after an acute physical activity may support this assumption (de Greeff et al., 2018). Though the ANT-C allows us to evaluate the functions of attention, this task required also important perceptual (visual) abilities, such as to identify the left or right direction of the target. Therefore, we speculate that, in our study, children limited their body mobility in order to stabilize their head and gaze and facilitate their perceptual activity (Stoffregen et al., 2000, 2007; Bonnet and Baudry, 2016).

### Incomplete Maturation of Orienting at 6 to 7Years?

No difference was found between the sitting and standing postures for the orienting performance, consistent with our initial hypothesis. A slightly larger orienting score was found when the children were free to move but this score remains small. Our results suggested that the body mobility condition does not improve the orienting function of children, which revolved around zero. Moreover, contrary to adults (Barra et al., 2015), information on the location of the upcoming target does not help children aged 6 to 7years to orient their attention better. Others studies showed a small orienting score in children at these ages (Mezzacappa, 2004; Ishigami and Klein, 2015), what suggests that the function of orienting may not be fully mature (Ishigami and Klein, 2015). These results are consistent with those of Mezzacappa (2004) which showed a development of the use of spatial information on the upcoming target between 5 to 7years.

### Improvement of Executive Control by Standing

Results showed a moderate improvement in executive control when the children stood compared to sitting. These results are consistent with some performance of adults (Rosenbaum et al., 2017; Smith et al., 2019). The executive control level is strongly influenced by arousal (Matchock and Toby Mordkoff, 2009; McConnell and Shore, 2011), which may be increased by a standing posture (Barra et al., 2015). This is consistent with physiological and neural modulations occurring when adopting a standing posture compared to a sitting posture (Tulen et al., 1999; Hennig et al., 2000; Caldwell et al., 2003). Easterbrook (1959) suggested that an optimal level of arousal may allow participants to be receptive of relevant information. This is in agreement with the increase in responsiveness to relevant information for a certain level of locus coeruleus activation (Aston-Jones and Cohen, 2005). Some authors have also suggested that a standing posture may induce a specific mental state in order to facilitate actions afforded by this posture (Thibault and Raz, 2016; Smith et al., 2019). In particular, an increase in information discrimination in the standing posture may support good and fast decision-making (e.g., to engage in fight or flight behavior; Smith et al., 2019). Furthermore, no improvement in executive control was found when children were free to move. In regard to alertness, we can speculate that too much freedom may not be optimal to perform this attention task, with the child requesting spatial reference points. More precisely, in regard to the studied visual cognitive task, 6- to 7-year-old children appear to need an environmental anchor that can hold their spontaneous body mobility.

### Limitations and Perspectives

This study has limitations. The relative small sample size and the variability between children, as frequently reported in this age range, may limit the generalization of the results. Further studies with increased number of children will be interesting to validate our results. It can also be noted that IQ of children was not evaluated in this study, especially to limit the experiment duration. Nevertheless, it was confirmed during interview with parents that none child had known cognitive, developmental, or motor disorder and that all children were in their appropriate grade level. Furthermore, the number of ANT-C trials for each body mobility condition was reduced as compared to the trials described by Rueda et al. (2004). We had to adjust the experimental design to maintain a sufficient level of engagement (Ishigami and Klein, 2015) and a reduced level of tiredness because of the young age of the children. Validation of the alerting effect and conflict effect suggests that children correctly performed the task in the three-body mobility conditions. In addition, the intra-individual variability in reaction time (Supplementary Figure S2) remained close enough to the values reported by (Lewis et al., 2018) for children of the same age, despite the smaller number of trials. Also, the free to move condition in this study did not allow us to precisely determine whether the results were related more to the absence of instruction on staying still or an increase in body mobility. Nevertheless, testing the influence of this ecological condition on attention networks is interesting because of its practical implications. We found no decline in alertness or executive control when children were free to move, supporting the idea that increasing the freedom of movement among children at school is not always detrimental. Finally, it could have been interesting to more precisely quantify the children’s movements. Nevertheless, a video analysis by two independent observers is an ecological way of evaluating the gross motor activity of children, easily applicable and reproducible in the practical field, and the inter-examiner agreement was correct in the present study.

As a perspective, it may be interesting to replicate the experiment by studying more than one age range with different schooling experience. This perspective is in accordance with the development of the functions of attention occurring between the ages of 6 to 12years, studies especially suggesting a turning point in middle childhood (Rueda et al., 2004) and in late childhood (Suades-González et al., 2017) for executive control and alertness, respectively. The level of spontaneous motor activity also seemed to undergo changes during childhood, with a gradual decrease of motor activity especially after the middle childhood (around 8years, (Eaton et al., 2001; Brocki et al., 2010). In that way, to compare the influence of body mobility on the functions of attention between the early, middle and late childhood seems interesting.

## Conclusion

This study suggests that attention performance in children aged 6 to 7years may depend on body posture, body mobility, and the attention function studied. Indeed, infants’ executive control was improved when they stood compared to sitting, while the performance of children for alertness and orienting when they seated, stood, and moved freely was close. In addition, children might also adapt their posture and their mobility according to the perceptual demand of the task and the devices used, that might influence their attention performance. Taking together, these results highlight the need to consider internal and environmental factors, such as the motor activity of children or the task studied, when their cognitive performance are evaluated. More broadly, it suggests that a better comprehension of the relationship between motor and attention activities may help to improve children learning and academic achievement.

## Data Availability Statement

The original contributions presented in the study are included in the article/Supplementary Material, and further inquiries can be directed to the corresponding author.

## Ethics Statement

The studies involving human participants were reviewed and approved by Comité de Protection des Personnes Sud-Est III (2017–010 B). Written informed consent to participate in this study was provided by the participants’ legal guardian/next of kin.

## Author Contributions

JR, J-MH, SC, and HC contributed to conception and design of the study. IH performed clinical examination for each child. JR, AP, and HC conducted the experiments, collected, and processed the data. JR, SC, and HC performed the statistical analysis. JR, HC, SC, and J-MH discussed the results and wrote the manuscript. All authors contributed to the article and approved the submitted version.

## Funding

This research is funded by the Université de Lorraine “Soutien à des Actions de Recherches – Crédits SC-UL 2017.”

## Conflict of Interest

The authors declare that the research was conducted in the absence of any commercial or financial relationships that could be construed as a potential conflict of interest.

## Publisher’s Note

All claims expressed in this article are solely those of the authors and do not necessarily represent those of their affiliated organizations, or those of the publisher, the editors and the reviewers. Any product that may be evaluated in this article, or claim that may be made by its manufacturer, is not guaranteed or endorsed by the publisher.

## Abbreviations

ANT: Attention network test
ANT-C: Attention network test for children
