# Supplementary material for: Body Mobility and Attention Networks in 6- to 7-Year-Old Children
Source: Front Psychol. 2021 Oct 27;12:743504. doi: 10.3389/fpsyg.2021.743504 (PMC8579035; doi:10.3389/fpsyg.2021.743504)
Supplement: Supplementary file 1 [file Presentation_1.PDF]

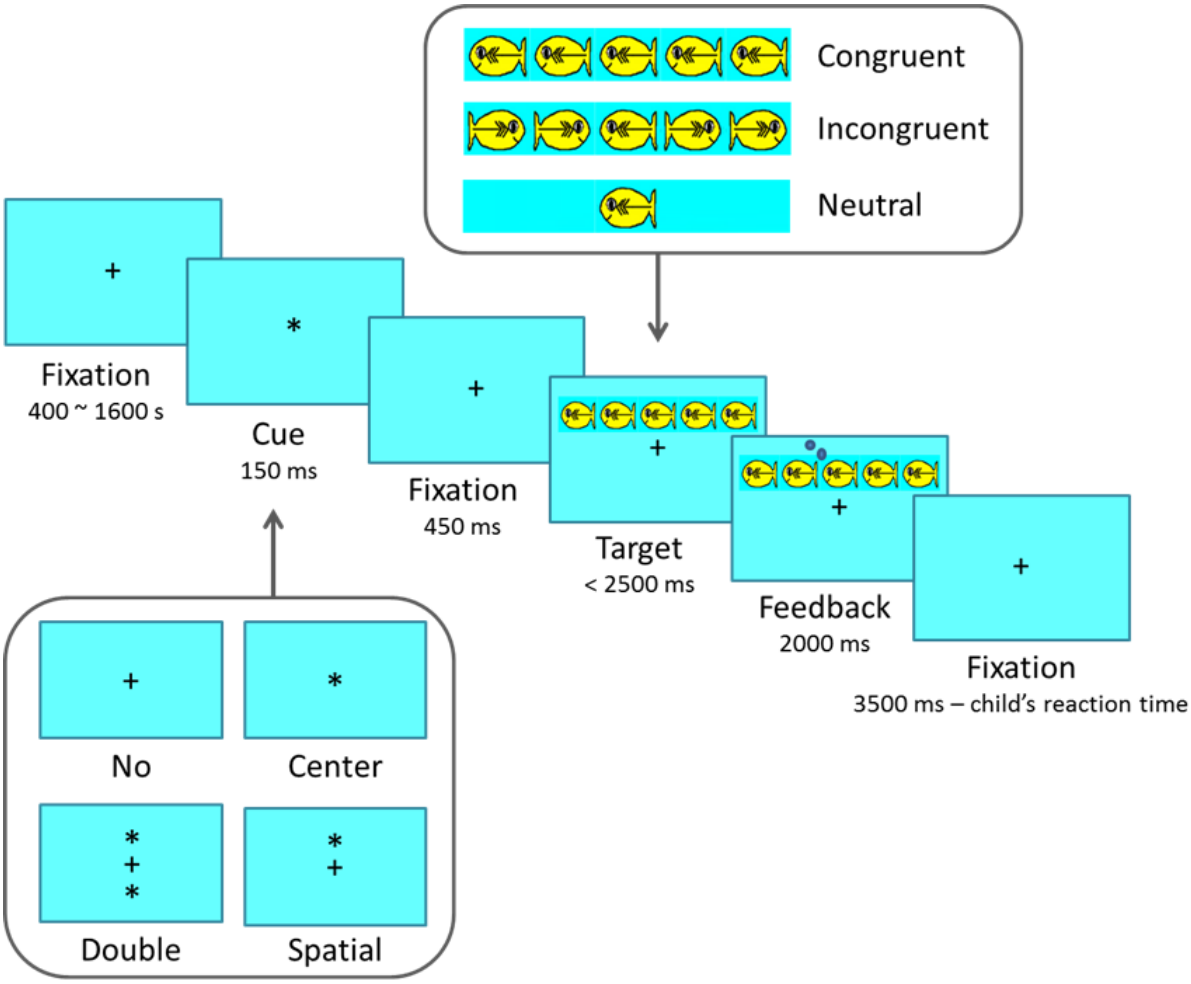

Supplementary Figure 1. Schematic of the Attention Network test for Children adapted from Rueda et al. (2004).
