# Supplementary material for: Body Mobility and Attention Networks in 6- to 7-Year-Old Children
Source: Front Psychol. 2021 Oct 27;12:743504. doi: 10.3389/fpsyg.2021.743504 (PMC8579035; doi:10.3389/fpsyg.2021.743504)
Supplement: Supplementary file 2 [file Presentation_2.PDF]

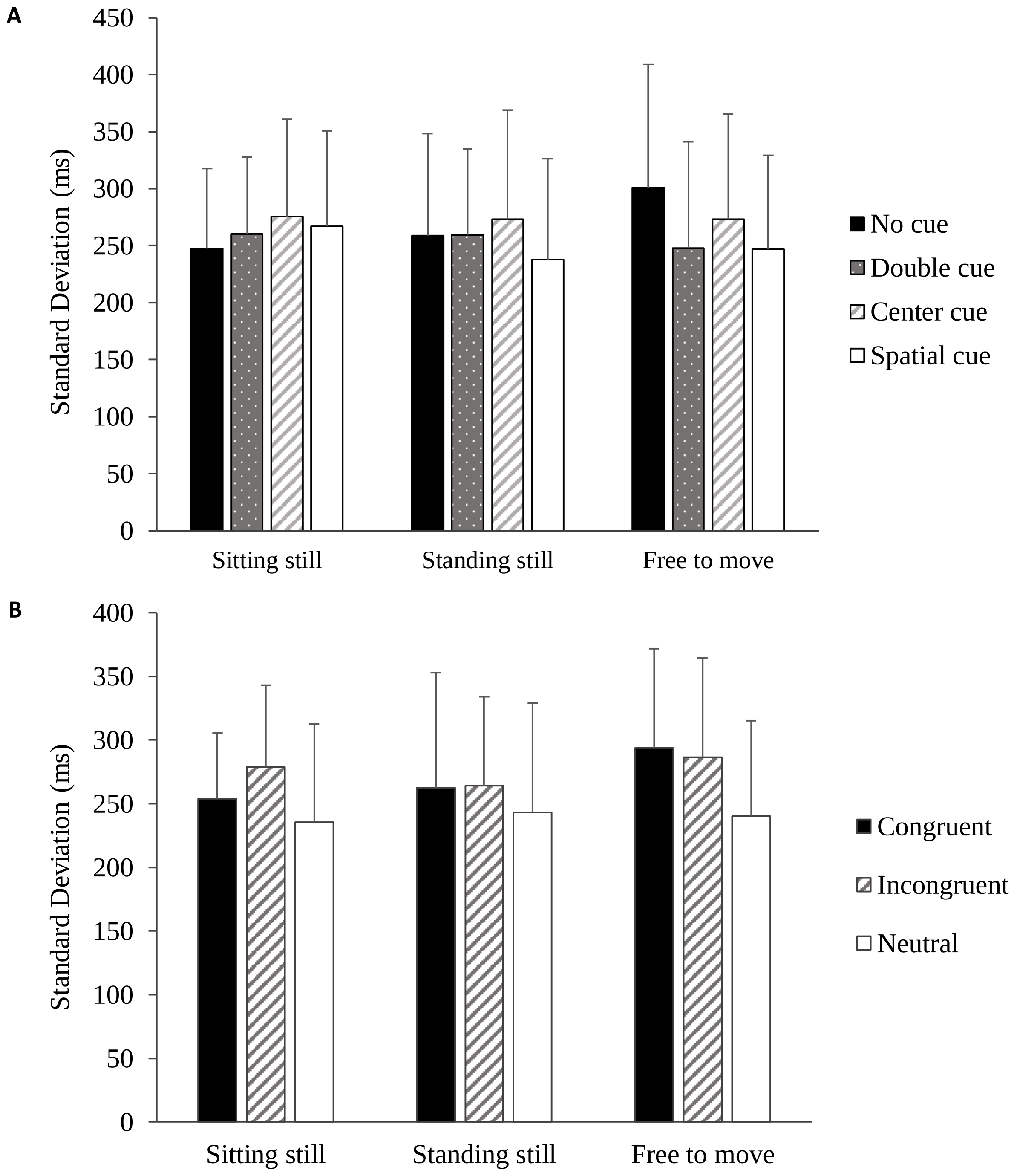

Supplementary Figure 2. Intra-individual variability of reaction times for cue (A) and target (B) in each body mobility condition. Error bars represent the mean absolute difference. Readers are referred to Lewis et al. (2018) to compare with values for children of similar age.
