# Supplementary material for: Body Mobility and Attention Networks in 6- to 7-Year-Old Children
Source: Front Psychol. 2021 Oct 27;12:743504. doi: 10.3389/fpsyg.2021.743504 (PMC8579035; doi:10.3389/fpsyg.2021.743504)
Supplement: Supplementary file 3 [file Data_Sheet_1.PDF]

Raw Data (*Attention Network Test*)

"NaN" values =
Commission/Omission Error [97,2% (*N = 174*) of "NaN" values]
OR
Reaction Time < 400 ms [2,8% (*N = 5*) of "NaN" values]

Child 1 =

		No	Double	Center	Spatial
Sitting	Congruent	965	829	871	947
Sitting	Congruent	928	1077	830	1733
Sitting	Congruent	851	555	731	719
Sitting	Congruent	1333	700	989	1073
Sitting	Incongruent	NaN	894	1378	1118
Sitting	Incongruent	1572	1248	1599	1507
Sitting	Incongruent	1171	1599	836	1344
Sitting	Incongruent	1993	789	982	976
Sitting	Neutral	822	729	2065	1294
Sitting	Neutral	638	728	737	833
Sitting	Neutral	NaN	615	742	598
Sitting	Neutral	912	1581	937	1127
Standing	Congruent	654	591	834	1024
Standing	Congruent	1104	979	1120	1325
Standing	Congruent	1024	1226	872	1261
Standing	Congruent	871	866	734	904
Standing	Incongruent	1515	1315	1787	1516
Standing	Incongruent	1355	2091	NaN	2040
Standing	Incongruent	1101	914	932	1108
Standing	Incongruent	759	NaN	1080	1000
Standing	Neutral	695	819	766	1320
Standing	Neutral	969	581	774	961
Standing	Neutral	906	956	739	750
Standing	Neutral	NaN	827	629	708
Free	Congruent	1325	1205	1523	1211
Free	Congruent	1311	1465	968	1324
Free	Congruent	1839	992	1020	945
Free	Congruent	728	1467	1188	1040
Free	Incongruent	2174	872	1405	1719
Free	Incongruent	1065	1211	1045	NaN
Free	Incongruent	1742	NaN	NaN	1553
Free	Incongruent	NaN	1389	1991	1193
Free	Neutral	1003	739	2069	805
Free	Neutral	916	1127	827	798
Free	Neutral	898	1100	2073	1145
Free	Neutral	1312	1012	841	986

Child 2 =

		No	Double	Center	Spatial
Sitting	Congruent	1122	1384	1559	1473
		1441	1919	1816	1343
		1611	1925	1656	1865
		1452	2010	1436	1179
	Incongruent	2064	2132	1566	1934
		1949	1859	NaN	1885
		1953	1677	1622	1323
		1861	1736	NaN	1662
	Neutral	1821	1141	1189	1158
		1165	1128	988	1058
		1295	1561	1288	1276
		1014	1277	1297	1363
Standing	Congruent	1508	805	975	811
		955	1005	1182	803
		1090	726	807	922
		890	1407	1198	1019
	Incongruent	929	972	1032	924
		1116	1444	1724	1023
		1082	1003	897	1300
		1405	1321	1312	1366
	Neutral	1140	922	800	747
		1213	782	1645	745
		1340	NaN	1692	1339
		1683	1133	1084	1100
Free	Congruent	1355	1149	841	1142
		807	765	672	773
		976	913	1033	896
		932	1343	1232	1527
	Incongruent	NaN	1426	1302	2055
		1076	1067	1449	1208
		1431	1231	998	1049
		1453	1053	1359	912
	Neutral	1136	969	838	1006
		2149	959	719	846
		872	788	919	833
		856	904	1009	1422

Child 3 =

		No	Double	Center	Spatial
Sitting	Congruent	948	1400	1319	1162
		861	789	1125	1511
		1138	822	845	860
		833	834	817	818
	Incongruent	1107	1131	1072	1266
		1010	1002	1339	998
		998	1304	994	996
		860	1005	1532	842
	Neutral	995	962	823	693
		726	1087	1056	887
		777	691	732	830
		1031	707	822	947
Standing	Congruent	NaN	893	1385	1695
		1142	1042	1133	883
		977	1803	1192	NaN
		NaN	1467	1061	1181
	Incongruent	NaN	NaN	1212	1452
		NaN	2177	NaN	1234
		1596	NaN	1361	NaN
		1315	1600	1289	1293
	Neutral	1409	NaN	1033	NaN
		1085	1349	1022	999
		1134	993	857	1718
		NaN	1225	1262	1034
Free	Congruent	1178	1164	1143	1171
		1098	959	1090	NaN
		869	751	889	NaN
		NaN	1581	NaN	653
	Incongruent	969	1122	1913	NaN
		NaN	1713	1159	831
		1370	1424	1181	NaN
		NaN	918	1136	985
	Neutral	1008	1196	726	970
		NaN	737	818	1058
		1129	1066	891	838
		NaN	990	684	544

Child 4 =

		No	Double	Center	Spatial
Sitting	Congruent	NaN	NaN	588	681
		NaN	592	756	528
		NaN	733	1051	1035
		924	911	765	588
	Incongruent	887	982	NaN	826
		1050	1377	1430	831
		894	1159	734	781
		1154	639	812	1075
	Neutral	925	780	905	NaN
		1157	761	850	556
		NaN	625	716	602
		979	988	790	710
Standing	Congruent	787	554	865	722
		787	685	490	609
		652	650	514	1031
		601	995	725	787
	Incongruent	821	1273	1063	682
		891	565	739	NaN
		824	NaN	622	700
		1032	589	933	639
	Neutral	583	523	676	551
		1067	517	880	580
		1119	664	723	523
		897	609	717	701
Free	Congruent	654	706	686	400
		763	622	630	526
		724	556	720	634
		796	528	618	419
	Incongruent	1365	750	1046	508
		849	742	869	907
		846	801	603	406
		890	709	750	NaN
	Neutral	953	666	777	525
		1590	654	674	521
		907	NaN	584	508
		896	NaN	606	719

Child 5 =

		No	Double	Center	Spatial
Sitting	Congruent	NaN	980	838	628
		782	799	891	580
		1265	737	715	895
		694	945	694	793
	Incongruent	1151	800	740	994
		917	775	797	924
		868	1616	584	811
		830	1030	1680	1471
	Neutral	840	1034	1005	1042
		1008	559	628	NaN
		882	821	747	590
		1672	633	1068	682
Standing	Congruent	1231	688	1107	861
		990	1403	NaN	549
		508	773	613	512
		640	1163	995	459
	Incongruent	994	NaN	757	868
		909	587	NaN	707
		947	NaN	537	473
		736	537	572	660
	Neutral	1051	679	608	635
		823	657	1046	709
		974	608	791	484
		701	524	775	846
Free	Congruent	751	787	1065	716
		1047	694	733	679
		708	568	1198	NaN
		678	654	695	401
	Incongruent	1302	993	1007	996
		925	702	1799	776
		951	806	781	993
		725	637	995	646
	Neutral	674	675	829	673
		1081	528	704	664
		658	875	737	724
		707	NaN	677	962

Child 6 =

		No	Double	Center	Spatial
Sitting	Congruent	884	715	783	605
		1295	822	809	815
		693	738	823	864
		901	1177	800	1017
	Incongruent	1076	1027	889	930
		1041	956	872	751
		729	587	1078	1153
		761	958	1561	869
	Neutral	774	870	839	509
		1150	838	1277	605
		854	1123	NaN	782
		1046	633	730	821
Standing	Congruent	1100	1283	774	879
		1260	696	2256	1208
		721	2229	1375	1200
		1093	1082	1377	737
	Incongruent	1722	1166	1426	1304
		920	879	1384	1031
		1218	1149	856	936
		1156	1013	1720	1330
	Neutral	1131	1156	582	671
		982	748	809	1026
		956	806	1650	937
		956	1547	704	1224
Free	Congruent	1016	751	861	800
		727	693	1939	708
		1161	1075	NaN	700
		1085	584	1004	1283
	Incongruent	779	1320	1386	NaN
		1090	NaN	NaN	949
		847	1110	1116	1050
		914	793	1769	1995
	Neutral	967	1065	979	943
		1409	1148	1035	1613
		1355	1019	958	1026
		NaN	892	674	1125

Child 7 =

		No	Double	Center	Spatial
Sitting	Congruent	1093	1875	1086	1278
		794	958	949	838
		902	823	1080	863
		2121	NaN	NaN	768
	Incongruent	1330	811	1021	1446
		1036	757	1019	911
		844	1084	766	815
		853	1050	987	1071
	Neutral	944	716	882	1221
		668	1197	982	815
		766	770	NaN	1018
		967	1000	812	940
Standing	Congruent	846	NaN	684	673
		1099	663	814	1173
		588	673	786	775
		1012	NaN	811	774
	Incongruent	930	700	1311	958
		702	1487	761	965
		1102	956	743	1049
		1315	793	889	955
	Neutral	871	760	NaN	878
		685	878	602	1270
		893	724	672	662
		934	593	773	873
Free	Congruent	1046	1171	1299	1015
		1272	828	947	861
		1532	1126	890	937
		849	848	NaN	1694
	Incongruent	1275	1002	1157	1064
		928	1301	1219	1041
		1160	1016	1503	1314
		1358	1164	701	995
	Neutral	1077	1093	1138	840
		1187	809	944	858
		811	1174	1437	830
		967	908	1161	721

Child 8 =

		No	Double	Center	Spatial
Sitting	Congruent	763	806	NaN	1238
		1120	1449	1293	759
		867	757	818	865
		784	858	1303	918
	Incongruent	1209	NaN	990	NaN
		1083	1655	NaN	NaN
		1014	1050	785	1314
		879	995	766	NaN
	Neutral	1187	672	647	1041
		926	1263	619	907
		859	1007	709	1238
		908	1029	1292	951
Standing	Congruent	937	619	817	986
		927	722	892	772
		766	1811	650	1213
		878	1146	698	NaN
	Incongruent	NaN	784	724	844
		1035	1008	NaN	655
		862	757	1277	548
		923	794	769	1380
	Neutral	756	937	861	762
		735	877	591	526
		1174	738	668	914
		776	778	864	914
Free	Congruent	806	578	714	973
		978	815	1024	NaN
		923	1663	582	1358
		1388	559	540	949
	Incongruent	776	1776	1076	671
		NaN	850	926	964
		1019	1095	887	683
		783	940	1101	644
	Neutral	531	644	859	778
		710	860	809	924
		854	590	804	807
		981	791	867	599

Child 9 =

		No	Double	Center	Spatial
Sitting	Congruent	931	967	833	617
		960	927	NaN	795
		874	996	830	1206
		1130	833	1302	1952
	Incongruent	993	699	758	979
		736	835	1297	707
		814	1553	1648	2092
		899	834	1458	990
	Neutral	714	747	773	892
		721	1000	914	809
		1034	1139	983	891
		1551	1175	1427	877
Standing	Congruent	1760	682	1353	1027
		1308	718	1149	1079
		813	837	714	1514
		NaN	796	1063	900
	Incongruent	1691	1021	814	2107
		1542	874	1927	1051
		987	1144	965	718
		1292	716	762	1519
	Neutral	967	864	NaN	1041
		857	732	885	1204
		721	760	768	875
		588	863	633	1056
Free	Congruent	632	819	1110	1371
		822	1597	1005	1079
		627	1223	1365	838
		810	1143	1106	892
	Incongruent	796	1066	715	804
		989	1086	725	810
		684	1489	967	1197
		1642	1032	588	NaN
	Neutral	771	1443	975	653
		845	653	1093	952
		1081	NaN	1039	1594
		787	951	947	1290

Child 10 =

		No	Double	Center	Spatial
Sitting	Congruent	1050	518	997	703
		1000	852	591	694
		1036	618	621	590
		672	613	630	482
	Incongruent	1025	595	1567	665
		794	571	1440	754
		681	586	784	593
		1225	610	758	590
	Neutral	648	847	608	608
		758	625	450	716
		764	563	634	638
		802	508	623	962
Standing	Congruent	655	851	654	839
		770	626	649	681
		637	691	NaN	688
		673	749	627	662
	Incongruent	772	979	935	807
		827	878	734	784
		NaN	696	584	546
		856	708	717	611
	Neutral	1285	631	703	894
		700	533	654	705
		623	474	618	602
		1379	836	639	582
Free	Congruent	821	1613	614	NaN
		767	403	704	841
	Incongruent	769	739	730	697
		916	788	581	676
	Neutral	750	592	779	854
		831	654	565	701

Child 11 =

		No	Double	Center	Spatial
Sitting	Congruent	1306	903	868	931
		NaN	963	668	1092
		1231	NaN	1027	804
		1721	794	931	977
	Incongruent	NaN	974	1179	1060
		1108	1003	816	888
		1179	862	1623	687
		NaN	1578	1357	NaN
	Neutral	1022	1962	1963	NaN
		1168	1029	604	973
		NaN	919	862	1053
		946	1287	839	1123
Standing	Congruent	1421	1434	697	888
		1396	899	900	901
		1439	794	664	814
		909	1055	862	742
	Incongruent	1818	1027	1060	1583
		NaN	1599	1108	1293
		NaN	937	829	NaN
		1078	882	1293	1044
	Neutral	1002	952	1172	1214
		NaN	NaN	987	976
		NaN	994	1997	552
		NaN	1023	857	1123
Free	Congruent	1240	543	1202	960
		2212	863	712	1353
		827	1698	1082	NaN
		748	739	939	841
	Incongruent	1005	2022	NaN	891
		2128	1877	1079	1156
		762	1155	740	1484
		1205	903	629	973
	Neutral	1410	1152	1688	2286
		882	894	764	942
		587	NaN	1087	870
		1139	1223	1218	1038

Child 12 =

		No	Double	Center	Spatial
Sitting	Congruent	949	856	754	1335
		1049	1241	763	888
		746	526	842	NaN
		703	685	687	1622
	Incongruent	860	978	1098	856
		1191	658	820	1095
		881	683	1191	1166
		999	949	526	679
	Neutral	682	690	740	721
		693	739	693	606
		765	544	646	597
		NaN	681	887	766
Standing	Congruent	2134	1200	780	1133
		2053	NaN	955	874
		845	630	702	911
		876	745	763	704
	Incongruent	1422	1140	1324	1030
		1051	1089	1223	967
		1595	944	791	782
		1100	853	862	920
	Neutral	1161	1074	888	833
		1599	860	897	996
		1216	566	510	551
		1020	518	604	961
Free	Congruent	1021	1129	825	1569
		1887	852	637	775
		863	NaN	788	876
		989	587	597	898
	Incongruent	823	863	1886	1026
		913	NaN	NaN	781
		1491	830	1218	NaN
		1250	952	705	622
	Neutral	938	654	675	661
		624	739	1122	989
		600	559	1562	684
		793	782	743	740

Child 13 =

		No	Double	Center	Spatial
Sitting	Congruent	687	577	606	842
		761	846	618	724
		954	476	605	668
		836	619	663	694
	Incongruent	1018	900	724	1164
		673	898	605	898
		882	613	628	651
		929	NaN	1086	595
	Neutral	651	579	629	773
		745	803	685	477
		643	638	737	706
		731	490	644	638
Standing	Congruent	913	723	756	598
		672	565	636	816
		675	443	547	656
		753	718	553	656
	Incongruent	834	971	561	707
		702	570	619	582
		647	675	613	492
		727	525	1019	742
	Neutral	634	583	579	614
		518	501	788	532
		848	458	529	625
		580	952	621	684
Free	Congruent	796	919	587	478
		699	710	559	663
		714	546	703	459
		946	583	767	682
	Incongruent	758	689	816	852
		745	689	712	692
		815	622	640	954
		662	650	862	1113
	Neutral	978	622	783	649
		686	915	775	474
		732	556	702	497
		924	549	583	565

Child 14 =

		No	Double	Center	Spatial
Sitting	Congruent	979	1260	1723	938
		1554	1011	917	973
		1646	1220	1822	929
		1076	1101	921	NaN
	Incongruent	1601	1663	1477	1758
		1653	NaN	1745	1207
		1315	1160	1016	1328
		1379	1952	970	980
	Neutral	1058	1961	809	1305
		NaN	900	1332	1456
		1291	915	1027	NaN
		NaN	1167	840	1434
Standing	Congruent	1529	1096	936	854
		1484	NaN	986	915
		NaN	1569	1287	1065
		1294	941	1859	1152
	Incongruent	1004	1484	1173	1387
		1121	1304	1938	1384
		1179	1643	908	1119
		1347	1255	1269	953
	Neutral	907	771	976	1001
		1099	796	1205	583
		911	1064	1126	926
		NaN	1118	842	1135
Free	Congruent	NaN	NaN	1407	943
		1959	801	1842	1392
		1105	1389	1793	552
		1882	1357	976	2208
	Incongruent	649	1318	969	1263
		1635	2032	1099	2289
		800	NaN	925	1037
		NaN	783	1838	1932
	Neutral	862	1864	1332	1173
		1334	1700	1145	1892
		NaN	1311	781	1112
		1573	2080	1181	1403

Child 15 =

		No	Double	Center	Spatial
Sitting	Congruent	1676	666	919	677
		805	778	882	1425
		886	607	849	681
		1039	853	817	743
	Incongruent	977	1357	1158	1498
		809	1660	807	567
		1005	889	646	1263
		745	667	904	893
	Neutral	NaN	618	740	700
		873	688	585	620
		906	811	593	737
		894	685	1110	1394
Standing	Congruent	1174	788	837	472
		1172	1094	709	676
		781	554	1195	693
		2207	771	1430	2066
	Incongruent	1070	1101	1612	840
		842	862	NaN	809
		1129	976	603	1240
		817	434	704	935
	Neutral	683	552	652	705
		894	916	1164	2241
		1241	515	1011	888
		864	1149	860	598
Free	Congruent	515	923	532	865
		975	409	581	966
		1067	NaN	631	833
		1150	862	770	726
	Incongruent	1020	NaN	458	881
		1846	706	896	1188
		929	830	970	1014
		819	791	1114	978
	Neutral	691	647	848	NaN
		938	774	1074	1087
		1833	726	424	864
		655	856	822	836

Child 16 =

		No	Double	Center	Spatial
Sitting	Congruent	798	947	1022	1605
		1049	1105	1005	800
		1492	906	NaN	858
		1068	1001	835	1387
	Incongruent	940	1734	1157	2098
		1218	1236	2150	1625
		1308	NaN	1099	1500
		1724	NaN	1304	801
	Neutral	NaN	1197	753	1027
		NaN	948	1782	1862
		924	1605	2004	825
		1218	1652	943	910
Standing	Congruent	515	923	532	865
		975	409	581	966
		922	1050	865	1143
		1489	1018	986	786
	Incongruent	1020	NaN	458	881
		1846	706	896	1188
		764	1695	1515	929
		1145	1095	807	1001
	Neutral	691	647	848	NaN
		938	774	1074	1087
		1665	915	2180	1384
		1985	742	1186	903
Free	Congruent	808	870	1395	1294
		1919	1007	1583	884
		1033	NaN	1045	855
		NaN	NaN	2004	878
	Incongruent	1888	1214	1132	1416
		1808	1412	665	1074
		1025	1437	1200	779
		1285	NaN	1058	990
	Neutral	767	960	NaN	1175
		852	633	1201	1413
		921	1184	1242	784
		1270	1152	774	NaN

Child 17 =

		No	Double	Center	Spatial
Sitting	Congruent	733	1010	1082	2092
		830	1146	732	1017
		918	731	901	747
		930	792	750	855
	Incongruent	1543	1158	705	1548
		926	1128	1034	1534
		1678	1159	975	1025
		1609	903	1003	944
	Neutral	641	948	1481	915
		1645	1859	747	1023
		1118	870	923	1082
		1052	1099	834	1371
Standing	Congruent	985	1215	1388	1022
		992	877	1190	657
		981	1507	1834	899
		829	1022	988	1042
	Incongruent	2064	1052	NaN	1330
		913	823	912	1668
		886	975	860	1850
		1032	1232	1092	1358
	Neutral	1808	898	1230	1568
		1051	1076	843	1213
		933	828	790	728
		945	778	875	1038
Free	Congruent	1087	754	914	1265
		1499	639	793	825
		1106	NaN	1085	1071
		1512	814	1536	768
	Incongruent	1459	1233	877	866
		1177	1578	1287	1016
		801	708	1492	892
		768	1346	987	NaN
	Neutral	1290	1209	1240	1189
		1124	823	1016	780
		945	1168	946	702
		973	1010	752	856

Child 18 =

		No	Double	Center	Spatial
Sitting	Congruent	734	440	834	618
		648	558	1017	567
		550	686	764	426
		1006	826	773	607
	Incongruent	723	732	942	714
		755	569	636	739
		652	1134	768	1290
		NaN	626	649	739
	Neutral	601	458	634	680
		719	445	566	820
		569	604	686	599
		1267	512	507	779
Standing	Congruent	906	1217	613	902
		1027	777	708	797
		1199	858	516	606
		764	798	604	623
	Incongruent	1051	NaN	879	1337
		NaN	NaN	1556	1049
		936	767	682	1012
		753	716	741	737
	Neutral	606	581	546	742
		1029	833	768	865
		904	785	575	856
		496	576	494	676
Free	Congruent	599	507	673	595
		630	928	733	561
		581	499	501	484
		1783	573	559	1012
	Incongruent	659	830	661	674
		673	NaN	814	907
		686	848	639	518
		611	587	577	NaN
	Neutral	NaN	566	762	553
		617	629	998	575
		694	840	465	486
		628	577	602	856

Child 19 =

		No	Double	Center	Spatial
Sitting	Congruent	1111	914	817	1678
		1221	875	1015	574
		1074	885	717	820
		1361	878	NaN	1376
	Incongruent	1086	1178	979	933
		837	909	NaN	813
		1177	967	1185	1431
		1556	1113	1066	1385
	Neutral	780	919	1102	859
		1516	1028	769	897
		832	880	619	596
		1117	NaN	989	734
Standing	Congruent	NaN	778	1187	955
		NaN	782	999	853
		1205	847	1127	808
		1227	842	853	1065
	Incongruent	1191	1104	943	1169
		1345	826	1001	939
		992	1207	994	764
		1269	1019	945	1008
	Neutral	1008	1070	766	NaN
		1260	925	NaN	819
		1169	775	781	834
		993	919	785	734
Free	Congruent	793	957	882	500
		678	795	794	1038
		1544	NaN	949	1091
		1762	1265	916	607
	Incongruent	702	763	447	887
		1199	786	617	817
		1436	869	797	995
		756	1018	851	906
	Neutral	1198	1088	1574	894
		1012	1107	969	1097
		1220	711	1655	799
		984	631	1063	1057

Child 20 =

		No	Double	Center	Spatial
Sitting	Congruent	994	872	558	450
		1455	610	672	409
		963	743	548	463
		809	525	929	707
	Incongruent	652	1368	801	820
		NaN	718	1738	NaN
		842	537	761	620
		949	780	641	874
	Neutral	941	479	786	624
		532	649	1100	539
		660	610	599	626
		813	561	1118	627
Standing	Congruent	723	812	643	529
		676	800	749	527
		685	624	726	654
		765	567	753	664
	Incongruent	779	NaN	1088	694
		761	675	875	815
		903	588	1140	536
		861	680	901	820
	Neutral	766	461	734	913
		658	598	497	586
		629	826	744	864
		698	621	683	516
Free	Congruent	NaN	718	1041	787
		876	563	749	647
		724	718	542	599
		587	556	859	611
	Incongruent	975	554	1040	690
		862	795	NaN	571
		1134	691	435	563
		680	1060	762	600
	Neutral	NaN	648	478	631
		892	583	783	612
		930	533	687	1010
		578	730	682	699

Child 21 =

		No	Double	Center	Spatial
Sitting	Congruent	1875	995	1074	910
		1211	1421	1383	1519
		1482	1213	1352	1159
		1017	NaN	1007	1670
	Incongruent	1494	1373	2027	1122
		2049	1111	NaN	1159
		NaN	1895	755	1621
		1291	945	1119	NaN
	Neutral	1732	1097	1692	NaN
		1128	1392	950	1134
		983	918	889	858
		1224	NaN	895	1352
Standing	Congruent	1206	1989	1457	1507
		1511	2111	1174	1386
		1171	1401	1201	1317
		1034	940	NaN	1130
	Incongruent	1836	1792	NaN	1612
		975	1207	1136	1311
		1223	2124	2107	1641
		NaN	1522	1457	1392
	Neutral	1117	1659	855	1303
		1950	1541	1281	1511
		1050	1907	1279	NaN
		1119	2094	791	1314
Free	Congruent	1245	1279	2108	1395
		2115	1987	1198	993
		1040	1409	1553	1195
		NaN	1232	1207	1313
	Incongruent	1871	1231	1675	1971
		2034	1211	1600	1326
		1590	1767	1846	NaN
		1445	1289	1109	1226
	Neutral	1415	1310	1395	1592
		1091	1104	1297	1439
		1867	999	1021	1301
		1370	1880	1119	1266
