# Supplementary material for: Body Mobility and Attention Networks in 6- to 7-Year-Old Children
Source: Front Psychol. 2021 Oct 27;12:743504. doi: 10.3389/fpsyg.2021.743504 (PMC8579035; doi:10.3389/fpsyg.2021.743504)
Supplement: Supplementary file 4 [file Data_Sheet_2.PDF]

Raw data (*Movements*)

**Exam_1_1**	**First observation of the first examiner**
**Exam_1_2**	**Second observation of the first examiner**
**Exam_2**	**Observation of the second examiner**

SITTING STILL

	Leg movement (s)			Foot movement (s)			Arm movement (s)			Hand movement (s)			Head movement (s)		
Child	Exam_1_1	Exam_1_2	Exam_2	Exam_1_1	Exam_1_2	Exam_2	Exam_1_1	Exam_1_2	Exam_2	Exam_1_1	Exam_1_2	Exam_2	Exam_1_1	Exam_1_2	Exam_2
C1	2	6	0	0	4	0	0	11	1	0	5	0	0	0	0
C2	0	0	0	0	0	0	0	0	0	0	0	0	0	0	0
C3	0	0	1	5	7	0	2	3	1	1	0	3	0	0	0
C4	0	0	0	0	0	0	0	0	1	2	2	0	0	0	0
C5	0	0	0	0	1	1	1	0	0	0	0	0	1	0	0
C6	0	0	0	0	0	0	1	0	0	0	0	0	0	0	0
C7	0	0	0	0	0	0	0	0	0	0	0	0	0	0	0
C8	0	0	0	0	0	0	6	6	3	7	0	0	0	7	0
C9	0	0	0	0	0	0	0	2	0	3	0	0	0	18	0
C10	0	0	0	0	0	0	0	2	3	0	1	0	0	1	0
C11	0	0	0	0	0	0	0	2	2	5	3	3	4	1	0
C12	0	0	0	1	2	0	0	1	0	24	13	24	0	0	3
C13	0	0	0	0	0	0	0	0	0	0	0	0	0	0	0
C14	0	0	0	0	0	0	3	4	4	1	1	0	0	0	0
C15	0	0	0	0	1	0	0	0	0	0	0	0	0	0	0
C16	5	23	6	28	18	0	20	18	22	0	2	0	2	5	2
C17	0	0	0	0	0	0	0	1	0	10	9	6	0	2	0
C18	0	0	0	1	1	0	1	1	0	0	0	1	0	0	0
C19	15	12	0	1	1	0	6	3	9	10	3	0	0	0	0
C20	2	5	0	7	5	3	0	0	0	19	28,5	22	2	1	0
C21	1	2	0	1	2	0	2	5	3	3	3	0	0	3	0

STANDING STILL

	Leg movement (s)			Foot movement (s)			Arm movement (s)			Hand movement (s)			Head movement (s)		
Child	Exam_1_1	Exam_1_2	Exam_2	Exam_1_1	Exam_1_2	Exam_2	Exam_1_1	Exam_1_2	Exam_2	Exam_1_1	Exam_1_2	Exam_2	Exam_1_1	Exam_1_2	Exam_2
C1	0	6	1	0	0	0	0	2	0	0	0	0	0	0	0
C2	0	0	0	0	0	0	0	0	0	0	0	0	0	1	0
C3	0	2	0	0	0	0	9	12	9	5	2	0	0	0	1
C4	0	0	0	0	0	0	0	0	0	5	3	0	0	0	1
C5	0	2	0	0	0	0	0	0	0	1	2	2	3	2	2
C6	0	0	0	0	0	0	0	4	2	54	27,5	44	0	0	0
C7	0	0	0	0	0	0	0	0	0	3	2	0	0	0	0
C8	0	0	0	0	0	0	5	2	0	2	0	0	0	4	0
C9	2	2	0	1	0	0	3	3	0	3	3	0	0	7	0
C10	0	0	0	0	0	0	0	0	0	0	0	0	0	0	0
C11	1	3	2	2	3	0	13	17	5	12	8	2	2	2	0
C12	1	2	1	0	0	0	0	1	1	22	3	2	0	1	1
C13	0	0	0	0	0	0	0	0	0	0	0	0	0	0	0
C14	0	0	0	0	0	0	0	0	0	1	2	2	1	1	0
C15	0	0	0	0	0	0	0	0	3	3	0	0	0	1	1
C16	0	0	0	0	0	0	16	16	8	0	0	0	0	0	0
C17	0	0	0	0	0	0	2	2	0	0	3	0	0	0	0
C18	0	2	0	1	0	0	0	0	0	2	0	0	0	0	0
C19	5	5	1	2	0	1	8	5	4	4	9	1	1	2	0
C20	0	0	0	0	0	0	1	2	2	4	3	4	0	0	0
C21	0	0	0	0	0	0	24	23	20	0	0	0	0	2	0

FREE TO MOVE

	Leg movement (s)			Foot movement (s)		
Child	Exam_1_1	Exam_1_2	Exam_2	Exam_1_1	Exam_1_2	Exam_2
C1	13	12	7	0	0	0
C2	0	0	0	1	1	0
C3	0	0	0	0	1	0
C4	6	6,5	30	3	0	7
C5	3	0	0	0	0	0
C6	26	13	15	5	3	6
C7	0	0	0	0	0	0
C8	0	0	0	0	0	0
C9	3	2	0	8	4	0
C10	0	0	0	0	2	0
C11	6	1	6	12	9,5	0
C12	0	0	0	0	0	0
C13	0	0	0	2	2	4
C14	0	0	0	1	1	0
C15	0	0	0	1	5	1
C16	90	128	110	142	62	50
C17	0	0	0	0	1	0
C18	0	3	0	1	0	0
C19	0	0	0	83	80,5	50
C20	0	0	0	6	5	1
C21	0	0	0	0	0	0

	Arm movement (s)			Hand movement (s)			Head movement (s)		
Child	Exam_1_1	Exam_1_2	Exam_2	Exam_1_1	Exam_1_2	Exam_2	Exam_1_1	Exam_1_2	Exam_2
C1	6	4	3	4	2	5	12	9	20
C2	0	2	2	2	3	1	1	1	0
C3	8	5	4	0	0	0	5	2	4
C4	1	0	0	13	6	4	0	0	0
C5	0	0	0	1	0	0	2	1	0
C6	0	1	5	107	30,5	50	0	0	0
C7	0	0	0	7	3	0	2	1	0
C8	2	2	6	4	4	0	0	2	0
C9	2	1	2	0	0	0	0	4	2
C10	0	0	0	0	3	0	0	1	0
C11	13	8	8	38	20	5	6	6	0
C12	3	4	2	11	6	1	1	1	0
C13	0	0	0	0	0	0	0	1	0
C14	11	11	12	0	0	0	0	0	0
C15	0	1	1	3	3	1	0	0	0
C16	32	41	9	0	0	0	0	0	0
C17	4	0	0	9	7	4	0	0	10
C18	0	0	0	3	3	0	1	1	0
C19	10	5	1	18	11,5	4	1	2	0
C20	0	0	0	19	10	25	0	0	0
C21	4	9	6	22	15	4	1	4	0

*For readers interested in calculating the **general amount of movements***

	Task duration (s)*		
Child	Sitting	Standing	Free
C1	288,43	286,248	288,593
C2	289,127	288,585	288,031
C3	288,035	291,088	288,548
C4	288,074	287,97	288,029
C5	288,009	287,967	288,009
C6	288,027	288,108	290,254
C7	288,549	288,012	288,017
C8	287,929	288,071	288,027
C9	287,932	289,16	289,183
C10	288,039	288,017	143,955
C11	290,998	290,986	288,463
C12	288,015	288,592	288,058
C13	288,026	288,002	287,972
C14	290,191	288,608	290,949
C15	288,046	288,546	288,517
C16	289,627	288,08	291,066
C17	288,007	287,974	288,046
C18	287,961	289,124	288,013
C19	287,972	288,557	287,944
C20	287,995	288,025	288,078
C21	290,745	289,752	289,136

Coefficient (% of body mass)**				
Leg	Foot	Arm	Hand	Head
0,17	0,0688	0,0536	0,0247	0,1444
0,16	0,0686	0,0535	0,0246	0,1457
0,15	0,0629	0,0514	0,0240	0,1746
0,16	0,0668	0,0528	0,0244	0,1555
0,16	0,0663	0,0526	0,0244	0,1583
0,15	0,0636	0,0517	0,0241	0,1712
0,16	0,0659	0,0525	0,0243	0,1600
0,15	0,0646	0,0520	0,0242	0,1666
0,16	0,0673	0,0530	0,0245	0,1529
0,15	0,0643	0,0519	0,0241	0,1652
0,15	0,0649	0,0521	0,0242	0,1679
0,16	0,0663	0,0526	0,0244	0,1618
0,15	0,0640	0,0518	0,0241	0,1580
0,16	0,0664	0,0526	0,0244	0,1692
0,16	0,0670	0,0529	0,0244	0,1577
0,15	0,0649	0,0521	0,0242	0,1544
0,16	0,0665	0,0527	0,0244	0,1652
0,16	0,0656	0,0524	0,0243	0,1572
0,16	0,0657	0,0524	0,0243	0,1616
0,15	0,0647	0,0520	0,0242	0,1609
0,16	0,0656	0,0523	0,0243	0,1659

* used to data normalisation on 1 min
see *Data Analysis - Movements of Children during the ANT-c*

** See Jensen, R. K. (1989). Changes in segment inertia proportions between 4 and 20 years. J. Biomech. 22, 529–536. doi:10.1016/0021-9290(89)90004-3
Note : The coefficients varied between children because the child age was used to calculate each segment mass
